# Supplementary material for: Emergent polar order in nonpolar mixtures with nonreciprocal interactions
Source: Proc Natl Acad Sci U S A. 2024 Dec 12;121(51):e2407705121. doi: 10.1073/pnas.2407705121 (PMC11665850; doi:10.1073/pnas.2407705121)
Supplement: Supplementary file 1 — Appendix 01 (PDF) [file pnas.2407705121.sapp.pdf]

Supporting Information for

Emergent polar order in non-polar mixtures with non-reciprocal interactions

Giulia Pisegna, Suropriya Saha, and Ramin Golestanian

E-mail: ramin.golestanian@ds.mpg.de

This PDF file includes:

Supporting text

Supporting Information Text

Extended versions of Eqs. 2 and 3 from the main text. We compute the deterministic dynamics of $\boldsymbol{J}$ and ρ directly from Eq. 1 of the main text. We report here the complete equations including all the non-linear terms up to second order in gradients.

The dynamics of the polar order parameter is governed by the following equation

$$\begin{aligned}\partial_t \boldsymbol{J} = &-\frac{2\alpha}{\rho^2}\boldsymbol{J}(\boldsymbol{\nabla}\cdot\boldsymbol{J}) - \frac{2\alpha}{\rho^2}(\boldsymbol{J}\cdot\boldsymbol{\nabla})\boldsymbol{J} + \frac{4\alpha}{\rho^3}\boldsymbol{J}(\boldsymbol{J}\cdot\boldsymbol{\nabla})\rho + |\boldsymbol{J}|^2\boldsymbol{J}\left(r-u|\boldsymbol{J}|^2\right) - \frac{2\boldsymbol{J}}{\rho}(1-3\rho^2)\nabla^2\rho \\ &- \frac{4K}{\rho^5}|\boldsymbol{J}|^2\boldsymbol{J}\nabla^2\rho + \frac{2K}{\rho^4}\boldsymbol{J}\nabla^2|\boldsymbol{J}|^2 + \frac{4K}{\rho^4}\boldsymbol{J}(\boldsymbol{J}\cdot\boldsymbol{\nabla})(\boldsymbol{\nabla}\cdot\boldsymbol{J}) + \left(\frac{2K|\boldsymbol{J}|^2}{\rho^4} - 1 + \rho^2\right)\boldsymbol{\nabla}(\boldsymbol{\nabla}\cdot\boldsymbol{J}) \\ &+ 4\rho(\boldsymbol{J}\cdot\boldsymbol{\nabla})\boldsymbol{\nabla}\rho + 2K(\boldsymbol{J}\cdot\boldsymbol{\nabla})\boldsymbol{\nabla}\left(\frac{|\boldsymbol{J}|^2}{\rho^4}\right) \\ &+ 8\boldsymbol{J}|\boldsymbol{\nabla}\rho|^2 + \frac{24K}{\rho^6}\boldsymbol{J}|\boldsymbol{J}|^2|\boldsymbol{\nabla}\rho|^2 - \frac{12K}{\rho^5}\boldsymbol{J}(\boldsymbol{\nabla}\rho\cdot\boldsymbol{\nabla}|\boldsymbol{J}|^2) + \frac{8K}{\rho^3}\boldsymbol{J}\left[\boldsymbol{J}\cdot\boldsymbol{\nabla}\left(\frac{\boldsymbol{J}}{\rho^2}\right)\cdot\boldsymbol{\nabla}\rho\right] \\ &- \frac{8K}{\rho^2}\boldsymbol{J}\left[\boldsymbol{J}\cdot\boldsymbol{\nabla}\left(\frac{\boldsymbol{J}}{\rho^3}\right)\cdot\boldsymbol{\nabla}\rho\right] - \frac{8K}{\rho^5}\boldsymbol{J}(\boldsymbol{\nabla}\cdot\boldsymbol{J})(\boldsymbol{\nabla}\rho\cdot\boldsymbol{J}) + \frac{2K}{\rho^4}\boldsymbol{J}(\boldsymbol{\nabla}\cdot\boldsymbol{J})^2 - \frac{8K}{\rho^6}\boldsymbol{J}(\boldsymbol{\nabla}\rho\cdot\boldsymbol{J})^2 \\ &+ \frac{2\boldsymbol{\nabla}\rho}{\rho}(\boldsymbol{\nabla}\cdot\boldsymbol{J}) + 4\rho^2\boldsymbol{\nabla}\rho\cdot\boldsymbol{\nabla}\left(\frac{\boldsymbol{J}}{\rho}\right) + 2K\rho^2(\boldsymbol{\nabla}\cdot\boldsymbol{J})\boldsymbol{\nabla}\left(\frac{|\boldsymbol{J}|^2}{\rho^6}\right) + 2K\rho^2\boldsymbol{\nabla}\left(\frac{\boldsymbol{J}}{\rho^2}\right)\cdot\boldsymbol{\nabla}\left(\frac{|\boldsymbol{J}|^2}{\rho^4}\right)\ .\end{aligned} \tag{S1}$$

When expanded in fluctuations around the ordered state, the last three lines will produce only non-linear (Renormalization Group irrelevant) terms, which can therefore be neglected. After introducing the following definitions in the remaining terms

$$\begin{gathered}\lambda_1 = 2\alpha/\rho^2, \quad \lambda_2 = 2\alpha/\rho^2, \quad \lambda_3 = 4\alpha/\rho^3, \\ m = \rho^4(r-u|\boldsymbol{J}|^2)/2, \quad r = 2(1-\rho^2)/\rho^4, \quad u = 2K/\rho^8, \\ \Gamma = 1-\rho^2-2m, \quad D_{lJ} = 4K/\rho^4, \quad D_{mJ} = 2K/\rho^4, \\ r_1 = 2(2m+5\rho^2-3)\nabla^2\rho/\rho, \quad P = 2\,\boldsymbol{J}\cdot\boldsymbol{\nabla}m,\end{gathered} \tag{S2}$$

we recover Eq. 2 of the main text.

The dynamics of the amplitude is governed by the following equation

$$\begin{aligned}\partial_t\rho + \frac{\alpha}{\rho}(\boldsymbol{\nabla}\cdot\boldsymbol{J}) = &-\mathcal{D}\nabla^2(1/\rho^2) + \frac{|\boldsymbol{J}|^2}{\rho^3}\left(1-\rho^2\right) - K\frac{|\boldsymbol{J}|^4}{\rho^7} - \nabla^2\left[\rho\left(1-\rho^2\right)\right] + 2K\frac{|\boldsymbol{J}|^2}{\rho^4}\nabla^2\rho + K\rho\nabla^2\left(\frac{|\boldsymbol{J}|^2}{\rho^4}\right) \\ &+4K\boldsymbol{\nabla}\left(\frac{\boldsymbol{J}}{\rho^2}\cdot\boldsymbol{\nabla}\rho\right)\cdot\left(\frac{\boldsymbol{J}}{\rho^2}\right) - \frac{4K}{\rho}\boldsymbol{J}\cdot\boldsymbol{\nabla}\left(\frac{\boldsymbol{J}\cdot\boldsymbol{\nabla}\rho}{\rho^3}\right) + \frac{2K}{\rho}\boldsymbol{J}\cdot\boldsymbol{\nabla}\left(\frac{\boldsymbol{\nabla}\cdot\boldsymbol{J}}{\rho^2}\right) \\ &-\frac{4K}{\rho^5}(\boldsymbol{\nabla}\rho\cdot\boldsymbol{J})^2 + \frac{K}{\rho^3}(\boldsymbol{\nabla}\cdot\boldsymbol{J})^2 + 2K(\boldsymbol{\nabla}\rho\cdot\boldsymbol{\nabla})\left(\frac{|\boldsymbol{J}|^2}{\rho^4}\right)\ .\end{aligned} \tag{S3}$$

Following the same reasoning as above, the last line will produce only non-linear irrelevant terms, which we do not retain in the main text. All the others terms are expanded in terms of irrelevant non-linear contributions and those contributing at the linear level. In the main text we keep only the latter. To recover Eq. 3, we introduce the following definitions

$$\lambda_4 = \alpha/\rho, \quad \beta = 1/\rho^3, \quad D_\rho = 5-3\rho^2+2m+\mathcal{D}/\rho^2, \quad \kappa = K/\rho^3, \quad w = 2K/\rho^3\ . \tag{S4}$$
